# Detection and Physicochemical Characterization of Membrane Vesicles (MVs) of *Lactobacillus reuteri* DSM 17938

**DOI:** 10.3389/fmicb.2017.01040

**Published:** 2017-06-13

**Authors:** Rossella Grande, Christian Celia, Gabriella Mincione, Annarita Stringaro, Luisa Di Marzio, Marisa Colone, Maria C. Di Marcantonio, Luca Savino, Valentina Puca, Roberto Santoliquido, Marcello Locatelli, Raffaella Muraro, Luanne Hall-Stoodley, Paul Stoodley

**Affiliations:** ^1^Department of Pharmacy, “G. d’Annunzio” University of Chieti-PescaraChieti, Italy; ^2^Center of Aging Sciences and Translational MedicineChieti, Italy; ^3^Department of Nanomedicine, Houston Methodist Research Institute, HoustonTX, United States; ^4^Department of Medical, Oral, and Biotechnological Sciences, “G. d’Annunzio” University of Chieti-PescaraChieti, Italy; ^5^National Center for Drug Research and Evaluation, Italian National Institute of HealthRome, Italy; ^6^Department of Biotechnological and Applied Clinical Sciences, University of L’AquilaL’Aquila, Italy; ^7^AlfatestLabCinisello Balsamo, Italy; ^8^Malvern Instruments Ltd.Worcestershire, United Kingdom; ^9^NIHR Wellcome Trust Clinical Research Facility, University Hospital Southampton NHS Foundation TrustSouthampton, United Kingdom; ^10^Department of Microbial Infection and Immunity, Centre for Microbial Interface Biology, The Ohio State University, ColumbusOH, United States; ^11^National Center for Advanced Tribology, Faculty of Engineering and the Environment, University of SouthamptonSouthampton, United Kingdom

**Keywords:** *Lactobacillus reuteri*, membrane vesicles (MVs), biofilm, nanoparticles, extracellular DNA, probiotics, commensal bacteria

## Abstract

Membrane vesicles (MVs) are bilayer structures which bleb from bacteria, and are important in trafficking biomolecules to other bacteria or host cells. There are few data about MVs produced by the Gram-positive commensal-derived probiotic *Lactobacillus reuteri*; however, MVs from this species may have potential therapeutic benefit. The aim of this study was to detect and characterize MVs produced from biofilm (bMVs), and planktonic (pMVs) phenotypes of *L. reuteri* DSM 17938. MVs were analyzed for structure and physicochemical characterization by Scanning Electron Microscope (SEM) and Dynamic Light Scattering (DLS). Their composition was interrogated using various digestive enzyme treatments and subsequent Transmission Electron Microscopy (TEM) analysis. eDNA (extracellular DNA) was detected and quantified using PicoGreen. We found that planktonic and biofilm of *L. reuteri* cultures generated MVs with a broad size distribution. Our data also showed that eDNA was associated with pMVs and bMVs (e_MVs_DNA). DNase I treatment demonstrated no modifications of MVs, suggesting that an eDNA-MVs complex protected the e_MVs_DNA. Proteinase K and Phospholipase C treatments modified the structure of MVs, showing that lipids and proteins are important structural components of *L. reuteri* MVs. The biological composition and the physicochemical characterization of MVs generated by the probiotic *L. reuteri* may represent a starting point for future applications in the development of vesicles-based therapeutic systems.

## Introduction

Membrane vesicles (MVs) are lipid bilayer structures of 20–500 nm in diameter containing various macromolecules, such as phospholipids, proteins, lipopolysaccharide (LPS), and nucleic acids (Brown et al., 2015). MVs represent a mechanism of communication between bacteria, and can modulate biological processes, such as biofilm development, quorum sensing, phage decoy, and horizontal gene transfer (Fong and Yildiz, 2015; Turnbull et al., 2016). Bacterial MVs can also deliver virulence factors to host cells in infections (Ellis and Kuehn, 2010). Various reports have demonstrated the functional roles and properties of outer membrane vesicles (OMVs) in Gram-negative bacteria (Mashburn-Warren et al., 2008; Schwechheimer and Kuehn, 2015; Lee et al., 2016), while the production of MVs from Gram-positive bacteria was demonstrated for the first time in 1990 (Dorward and Garon, 1990; Brown et al., 2015). More recent papers showed the production of MVs from Gram-positive bacteria, such as *Staphylococcus aureus*, *Listeria monocytogenes*, *Streptococcus pneumoniae* and *Clostridium perfringens* (Gurung et al., 2011; Lee et al., 2013; Brown et al., 2014, 2015; Olaya-Abril et al., 2014).

Although there is increasing information about the production of MVs from Gram-positive, and negative pathogens, few data are available on MVs generated by probiotic bacteria. Furthermore, the mechanism of communication between probiotic bacteria and host is unclear (Mayer, 2011; Collins et al., 2012; Forsythe and Kunze, 2013; Al-Nedawi et al., 2014). Commensal bacteria can modulate the physiological mechanisms of immune, endocrine, and nervous systems; however, only a few commensal bacteria are in direct contact with the intestinal epithelium, and many are located away from the epithelium in the adherent mucus layer (Al-Nedawi et al., 2014). It has been hypothesized that MVs play a role in modulating communications between commensal bacteria of gastrointestinal lumen and central nervous system (CNS), thus supporting the hypothesis of the microbiome–gut–brain axis (Gareau et al., 2011; Bravo et al., 2012; Dinan et al., 2013; Forsythe and Kunze, 2013; Al-Nedawi et al., 2014). Although the activity of probiotics is strain specific; generally, commensal bacteria play a protective role toward the host by inhibiting the colonization of pathogens, and modulating the host immune response in the gastrointestinal tract (Jones and Versalovic, 2009). Furthermore, some bacterial species can attenuate depression and chronic fatigue syndrome (Dinan et al., 2013). MVs production by *Lactobacillus rhamnosus* JB – 1 represents an important mechanism of communication between commensal bacteria and the host (Al-Nedawi et al., 2014). López et al. (2012) also demonstrated that *Bifidobacterium bifidum* LMG13195 MVs could activate the maturation of dendritic cells, and induce a regulatory response of T cells.

*Lactobacillus reuteri* colonizes the gastrointestinal tract of vertebrates (Hou et al., 2015). The probiotic *L. reuteri* forms biofilm *in vitro*, generates immunomodulatory factors (Jones and Versalovic, 2009), and demonstrates a specific activity against infantile colic, eczema and *Helicobacter pylori* colonization (Abrahamsson et al., 2007; Imase et al., 2007; Jones and Versalovic, 2009). Various studies have demonstrated differences in OMVs generated in the biofilm and planktonic phenotypes. For example, OMVs produced in the planktonic and biofilm phenotypes of *Pseudomonas aeruginosa* exhibited different proteomes (Toyofuku et al., 2012; Park et al., 2015) while, OMVs produced by *H. pylori* in the biofilm phenotype had higher amounts of eDNA compared to planktonic OMVs suggesting a structural role of OMV-associated nucleic acid in the biofilm (Grande et al., 2015).

The aim of the present work was to detect and physicochemical characterize the MVs generated by *L. reuteri* in the planktonic and biofilm phenotypes. The study and the characterization of MVs may support the design of vesicles-based therapeutic systems.

## Materials and Methods

### Bacterial Strain and Media

*Lactobacillus reuteri* DSM 17938, a commercially available probiotic strain, which originated from ATCC 55730 (Rosander et al., 2008), was used in the study. *L. reuteri* DSM 17938 is beta-lactam, tetracycline, and lincosamide free, and does not provide any resistance determinants. The strain was plated on deMan, Rogosa, Sharpe Agar (MRS) (Oxoid Limited, Hampshire, United Kingdom), and incubated at 37°C for 24 h in an anaerobic atmosphere (O_2_ < 0.1% and 7% < CO_2_ < 15%) (Anaerogen Pak Jar, Oxoid Ltd).

### Biofilm Formation Assay

Bacteria were harvested in MRS broth (Oxoid Ltd) and incubated overnight at 37°C in anaerobic atmosphere under shaking at 90 rev min^-1^. After incubation, each broth culture was adjusted to an optical density at 600 nm (OD_600_) of 0.10 corresponding to 8.38 × 10^6^ CFU/ml and inoculated into both 90 mm diameter Petri dishes (Corning Incorporated, Corning, NY, United States) and 35 mm diameter Petri dishes (Ibidi GmbH, Planegg, Germany). Bacteria were incubated at 37°C in anaerobic atmosphere, without shaking, for 24 h. After incubation, non-adherent cells were harvested, while the biofilms were rinsed with calcium and magnesium Phosphate Buffered Saline free (PBS; pH 7.2). The biofilm cultures, inoculated in 35 mm Petri dishes, were used to test biofilm formation by SYTO 9 staining and Confocal Laser Scanning Microscopy (CLSM) analysis. The biofilm cultures in 90 mm Petri dishes were used for Scanning Electron Microscopy (SEM) analysis and MVs extraction. In particular, for the MVs extraction, biofilms were scraped, added to 20 ml of PBS and treated for the biological and physicochemical characterization.

### Evaluation of Biofilm Formation and MVs Production

*Lactobacillus reuteri* biofilms were developed as previously described on 35 mm Petri dishes and examined for evaluating the biofilm formation by CLSM using SYTO 9 staining according to the manufacturer’s instructions (Life Technologies, Carlsbad, CA, United States). The samples were visualized using a Zeiss LSM510 META confocal system (Jena, Germany) connected to an inverted Zeiss Axiovert 200 microscope equipped with a Plan Neofluar oil–immersion objectives (63×/1.4 and 100×/1.45 NA). SYTO 9 staining (green fluorescence) was excited using an argon laser with an excitation wavelength of 488 nm and set at 6% of power. All experiments were performed at room temperature, and each Petri dish was exposed to the laser for no more than 10 min.

Membrane vesicles production was analyzed using SEM. Briefly, 24 h after incubation, the biofilms and their corresponding planktonic phenotypes were centrifuged for 20 min at 4000 × *g* at 4°C, washed twice with PBS, loaded on glass coverslips (12 mm in diameter), fixed for 1 h at room temperature with 2.5% (v/v) glutaraldehyde in a 0.2 M cacodylate buffer (pH 7.4). After three washes in the same buffer, the samples were post-fixed with 1% (w/v) OsO_4_ for 1 h, dehydrated through an ethanol gradient (Stringaro et al., 2014), critical point dried in CO_2_ and sputter coated with gold. The samples were examined by scanning electron microscope FEI Quanta Inspect FEG (FEI, United States).

### MVs Extraction

The MVs extraction from *L. reuteri* was performed on biofilm and planktonic cultures as previously reported (Grande et al., 2015). Briefly, *L. reuteri* biofilms, scraped and suspended in PBS, were centrifuged (5000 × *g*, 20 min at 4°C) and the resultant supernatants were filtered through 0.22 μm cellulose membrane filters (Corning, United States). Two hundred microliters of both planktonic and biofilm filtrates were spread on MRS agar and incubated at 37°C on anaerobic conditions to confirm the total absence of *L. reuteri* colonies. The samples were further purified using a Beckman coulter Optima XL – 100K ultracentrifuge (Beckman coulter, United States) at 50000 rpm, for 2 h at 4°C, washed with PBS and ultra-centrifuged for the second time (50000 rpm, 2 h at 4°C). The pellets were then dissolved in 200 μl PBS and stored both at -80 and 4°C. To visualize pMVs and bMVs, samples were negative stained and analyzed through a Transmission Electron Microscopy (TEM). Briefly, a drop of vesicles suspension was placed onto a formvar–carbon–coated grid (Electron Microscopy Sciences, Hatfield, United Kingdom), and negatively stained with phosphotungstic acid solution (1% v/v). Samples were then analyzed with a Philips 208 TEM (2–120 kV, 480,000×) (FEI, Eindhoven, Netherlands).

### pMVs and bMVs Enzymatic Treatment

The biochemical composition of the pMVs and bMVs was carried out by treating MVs with DNase I, Proteinase K and Phospholipase C. The experiments were slight modified and performed as previously reported (Chebotar et al., 2013). Briefly, 40 μl of each sample were treated with 10 μl DNase I (Sigma–Aldrich, St. Louis, MO, United States), Proteinase K (Qiagen GmbH, Hilden, Germany), and Phospholipase C type I (Sigma–Aldrich). The pMVs and bMVs were incubated for 15 min, at room temperature, with DNase I; 2 h, at 37°C, with Proteinase K; and 10 min, at 37°C, with Phospholipase C, respectively. The samples were subsequently treated for TEM analysis as previously reported (MVs extraction).

### Physicochemical Characterization of pMVs and bMVs

The average size, size distribution and zeta (Z)-potential of pMVs and bMVs were performed by using Dynamic Light Scattering (DLS) analysis as previously reported (Celia et al., 2013; Marianecci et al., 2013). Briefly, pMVs and bMVs were firstly filtered through 0.22 μm cellulose filter membrane, and further analyzed using a Zetasizer Nano ZS with a 4.5 mW laser diode, operating at 670 nm as a light source, and the scattered photons detected at 173°. A third order cumulative fitting autocorrelation function was applied to measure average size and size distributions. The analysis was carried out according to the following instrumental set up: (i) a real refractive index of 1.59; (ii) an imaginary refractive index of 0.0; (iii) a medium refractive index of 1.330; (iv) a medium viscosity of 1.0 mPa × s; and (v) a medium dielectric constant of 80.4 (Kirui et al., 2015). The pMVs and bMVs were pre-filtered (0.22 μm polypropylene membrane filter, Whatman Inc., Clifton, NJ, United States), and suitable diluted (RNase free water), before the analysis, to avoid multiscattering phenomena. DLS was further used to measure the polydispersity index (PDI) of the particle distribution. PDI is a measure of breadth of the distribution with PDI < 0.4 for a narrow size distribution, and >0.4 for a broad distribution of particles (Cosco et al., 2012; Paolino et al., 2013).

The Z-potential was used to measure the membrane charge of pMVs and bMVs. The analysis was performed using a Doppler laser anemometry function using the Zetasizer Nano ZS. The Z-potential was related to the electrophoretic mobility. A Smoluchowski constant F (Ka) of 1.5 was applied during the analysis. The apparatus consists of the following set up: a He/Ne laser doppler anemometry (633 nm) with a nominal power of 5.0 mW. The electrophoretic mobility values, which were measured simultaneously by the Zetasizer Nano ZS were used to corroborate the Z-potential values, as previously reported (Wolfram et al., 2014). Results are reported as the average ± standard deviation of ten independent replicates.

### Nanoparticle Tracking Analysis (NTA)

The physicochemical characterization of bMVs and pMVs was also investigated by using the Nanoparticle tracking analysis (NTA). pMVs and bMVs were extracted as herein reported (MVs extraction), suitable diluted with PBS, and directly tracked using the NanoSight NS300 system (NanoSight^TM^ technology, Malvern, United Kingdom). The analysis was carried out according to the following instrumental set up: (i) a laser beam of 488 nm (blue); (ii) and a high-sensitivity sCMOS camera. Videos were collected and analyzed using the NTA software (version 3.0) at 30 frames per second (fps), capturing a video file of the particles moving under Brownian motion. The software tracks many particles individually and using the Stokes–Einstein equation calculates their hydrodynamic diameters. Multiple videos of 60 s duration were recorded generating replicate histograms that were averaged. The final concentration of pMVs and bMVs was 4.04 × 10^10^ (particles/ml) and 2.22 × 10^10^ (particles/ml), respectively.

### Detection and Quantification of eDNA and proteins associated with MVs

The extracellular DNA (eDNA) associated with pMVs and bMVs (e_MVs_DNA) was detected and quantified by using Quant-iT^TM^ PicoGreen dsDNA assay kit (Life Technologies) according to manufacturer’s instructions. The PicoGreen can label both extra-vesicular DNA and eDNA associated with the MVs (e_MVs_DNA). DNase I treatment was performed to remove of any extravesicular eDNA that might be present. All measurements were carried out as three independent experiments. Protein concentrations of *L. reuteri* MVs were quantified by using the bicinchoninic acid (BCA) Protein Assay Kit (Pierce, Rockford, IL, United States). As previously reported (Mincione et al., 2014).

120 and 80 μg of proteins were extracted from pMVs and bMVs samples, respectively, and the e_MVs_DNA yield was normalized by using 10 μg of proteins.

### Statistical Analysis

Results represent the mean ± standard deviation (SD) or standard error of the mean (SEM). The statistical analysis of data was performed using the *t*-test; while the statistical significance of data was set at *p* ≤ 0.05.

## Results

### Biofilm Characterization

**FIGURE 1:**
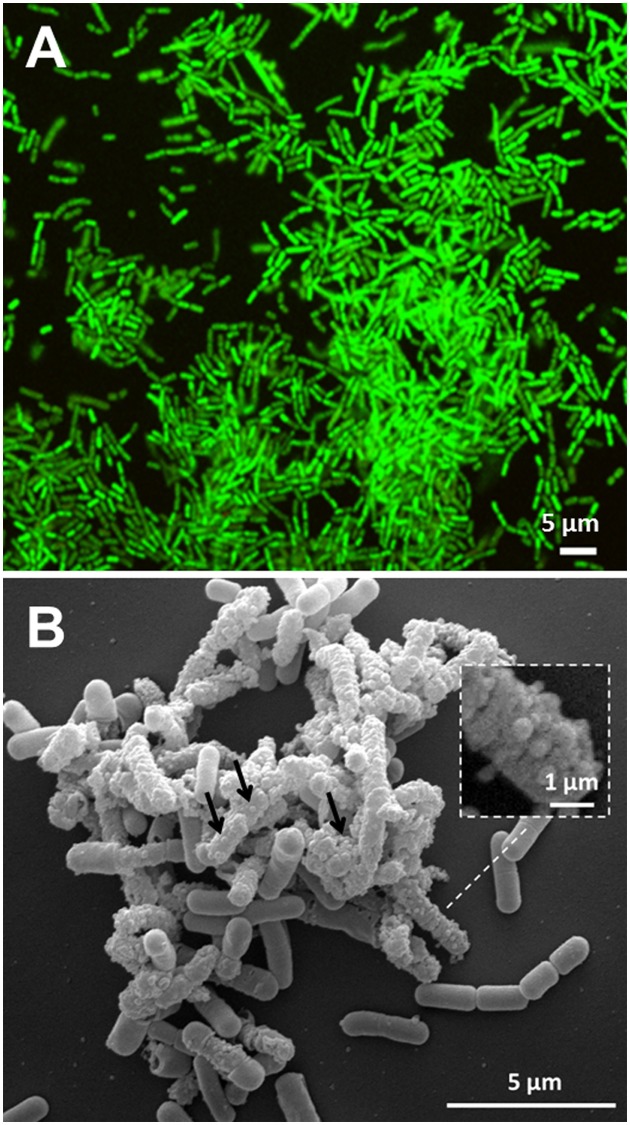
*Lactobacillus reuteri* DSM 17938 biofilm development after 24 h of incubation. **(A)** CLSM image of biofilm stained with SYTO 9; **(B)** representative SEM image of biofilm where vesicles were attached to cells (arrows). Magnification of vesicles blebbing from the bacterial surface (Square insert). A representative example of five independent experiments.

*Lactobacillus reuteri* DSM17938 formed biofilms of between 7–12 μm thick after 24 h growth (**Figure 1A**) that showed similar structures to those previously reported by Jones and Versalovic (2009) for *L. reuteri* ATCC 55730 biofilms.

Scanning Electron Microscopy analysis revealed that many cells in the biofilm showed MVs blebbing from them (**Figure 1B**).

### MVs Isolation: Ultrastructural and Physicochemical Characterization

#### Ultrastructural Analysis of pMVs and bMVs by TEM

**FIGURE 2:**
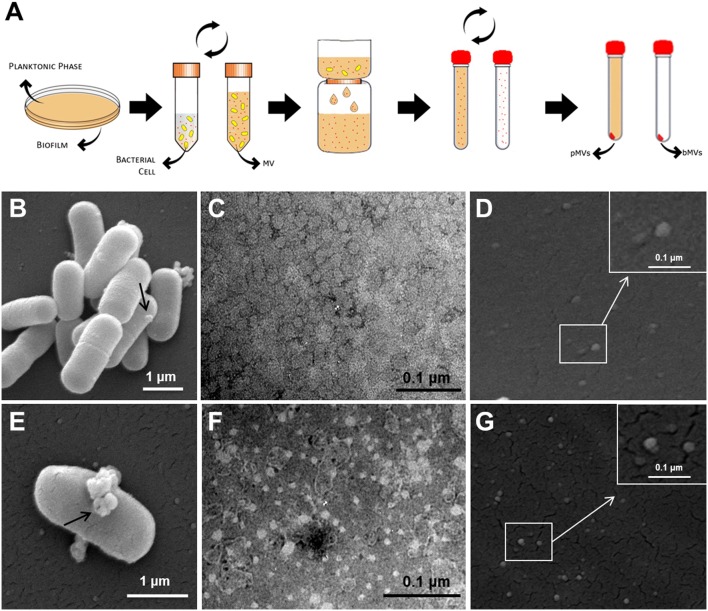
*Lactobacillus reuteri* DSM 17938 membrane vesicles isolation from planktonic and biofilm phenotypes. pMVs and bMVs isolation procedure **(A)**; SEM image of a biofilm sample containing *L. reuteri* cells, which generate extracellular vesicles (arrow) **(B)**; SEM image of a planktonic cell producing multiple vesicles (arrow) **(E)**; Negative staining analysis of bMVs **(C)** and pMVs **(F)**; vesicles released from *L. reuteri* biofilm cells **(D)** and planktonic cells **(G)** detected by SEM. Magnification of MVs (Square insert). Representative images of six independent experiments.

Pellets of bMVs and pMVs showed different structures and features. The pellet of bMVs was transparent and had a gel-like structure, while the pellet of pMVs was dense and white. TEM showed a broad particle distribution (50–150 nm), and polymorphic structures (**Figures 2C,F**); while SEM showed single vesicles blebbing from planktonic and biofilm phenotypes (**Figures 2D,G**). The SEM analysis further showed the production of multiple aggregated vesicles by single cells (**Figures 2B,E**).

#### Physicochemical Characterization of pMVs and bMVs by DLS

**FIGURE 3:**
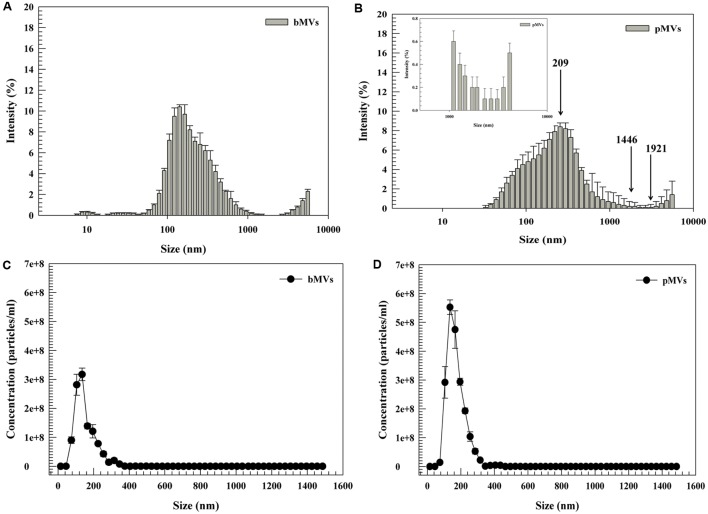
Physicochemical characterization of *L. reuteri* DSM 17938 through DLS and Nanosight NS300 system (NTA). The DLS histogram analysis of bMVs and pMVs are shown in **(A,B)** respectively. bMVs and pMVs had a bimodal distribution of nanovesicles. The nanoparticle tracking distribution of bMVs and pMVs is shown in **(C,D)**. Figures are representative of ten independent replicates for both DLS and NTA analysis. The size distribution represents the wide distribution of vesicles. The size distribution was calculated as intensity (%) using the multimodal distribution of software (Malvern Instruments Ltd.). Arrows show the mean peaks of particles at 209 nm (Peak 1), 1446 nm (Peak 2), and 1921 nm (Peak 3) as herein reported in Sub-section “Physicochemical Characterization of pMVs and bMVs by DLS” of Section “Results.” The error bars represent the standard deviation.

Dynamic Light Scattering results demonstrated that bMVs and pMVs were spherical in shape (**Figures 2C,F**), and had a broad size distributions (**Figures 3A,B**) with a PDI over 0.45 in planktonic and biofilm phenotypes, respectively (Supplementary Figure S1). bMVs had average sizes in the range from 6.0 nm to 4 μm (**Figure 3A**); conversely, pMVs had average sizes in the range from 210 nm to 2 μm (**Figure 3B**). Large particles of MVs may represent aggregated vesicles detected by SEM analysis as herein reported (**Figures 2B,E**).

Dynamic Light Scattering analysis demonstrated that bMVs had three different peaks at 236 nm (Peak 1; 92.4%), 3896 nm (Peak 2; 6.4%), and 6.03 nm (Peak 3; 1.2%) (**Figure 3A**); conversely pMVs have three different peaks at 209 nm (Peak 1; 78.3%), 1446 nm (Peak 2; 18.9%), and 1921 nm (Peak 3; 2.8) (**Figure 3B**). The resulting peaks of DLS analysis were different comparing bMVs and pMVs. These data corroborated the PDI results, which measured 0.44 (SD ± 0.08) and 0.5 (SD ± 0.11) for bMVs and pMVs, respectively (Supplementary Figure S1).

Nanoparticle tracking analysis facilitated directly visualizing the size and measuring the concentration of nanoparticles in liquid suspension, thus overcoming the limited information about the particle size distribution profile of polydisperse particles. The NTA analysis demonstrated that 90% of particles had an average size of 236 nm (#@ 315 particles/ml) for bMVs (**Figure 3C**), and 210 nm (#@ 415 particles/ml) for pMVs (**Figure 3D**), respectively. Differences for the number of particles counted through NTA analysis depended on the native concentration of bMVs (1.1 × 10^9^ particles/ml), and pMVs (2.02 × 10^9^ particles/ml) that were extracted from *L. reuteri*. Furthermore, NTA analysis further showed that bMVs had a narrow size distribution (**Figure 3C**); while pMVs were more broadly distributed (**Figure 3D**) suggesting the presence of larger particles under planktonic conditions, which may have aggregated from individual pMVs (**Figure 3D**).

Biofilm membrane vesicles (Supplementary Movie 1 and Table S1) and pMVs (Supplementary Movie 2 and Table S1) showed different distribution of particles in liquid suspensions that moved under Brownian motion in a polydispersity sample. The analysis was carried out using PBS as solvent flow, the flow rate was automatic setted through the software as previously reported (Dragovic et al., 2011; György et al., 2012).

The Z-potential values and electrophoretic mobilities showed negative values for both bMVs and pMVs. The net negative charge of biofilm and planktonic phenotypes was consistent with a cell wall charge. The Z-potential values were -13.4 mV (SD ± 1.1) with an electrophoretic mobility of -1.1 (μm × cm)/Vs (SD ± 0.08) for bMVs; and -39.8 mV (SD ± 1.5) with an electrophoretic mobility of -3.12 (μm × cm)/Vs (SD ± 0.12) for pMVs, respectively (Supplementary Figure S2). These values were significantly different (*p* < 0.001).

### Detection and Quantification of eDNA and Proteins Associated with MVs

**FIGURE 4:**
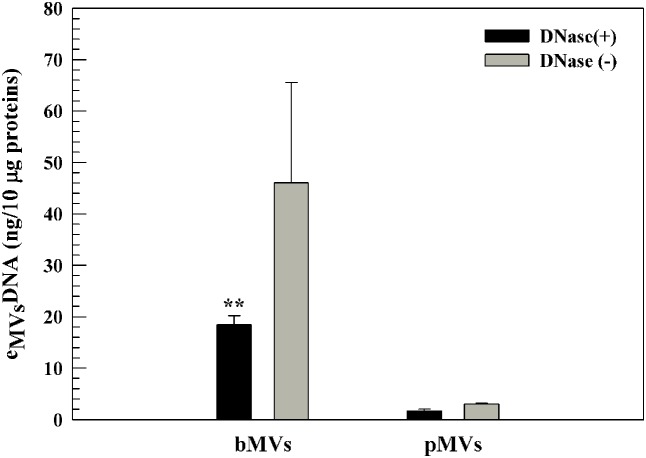
Detection and quantification of e_MVs_DNA associated to biofilm and planktonic phenotypes. eDNA of bMVs and pMVs treated with or without DNase I by using PicoGreen assay. The e_MVs_DNA content was normalized by using 10 μg of proteins. ^∗∗^*p* ≤ 0.01 compared to pMVs. The results are the mean of three different experiments ± SD as triplicates.

The e_MVs_DNA was detected and quantified by using Quant-iTTM PicoGreen dsDNA assay kit. The concentration of e_MVs_DNA associated with biofilm and planktonic phenotypes of *L. reuteri* DSM 17938 demonstrated that bMVs contained more e_MVs_DNA than pMVs in both DNase I treated (*p* ≤ 0.01) and untreated samples (**Figure 4**).

However, the protein concentration was higher for pMVs than bMVs (*p* ≤ 0.001) (Supplementary Figure S3).

### Analysis of MVs Composition by Enzymatic Treatment

**FIGURE 5:**
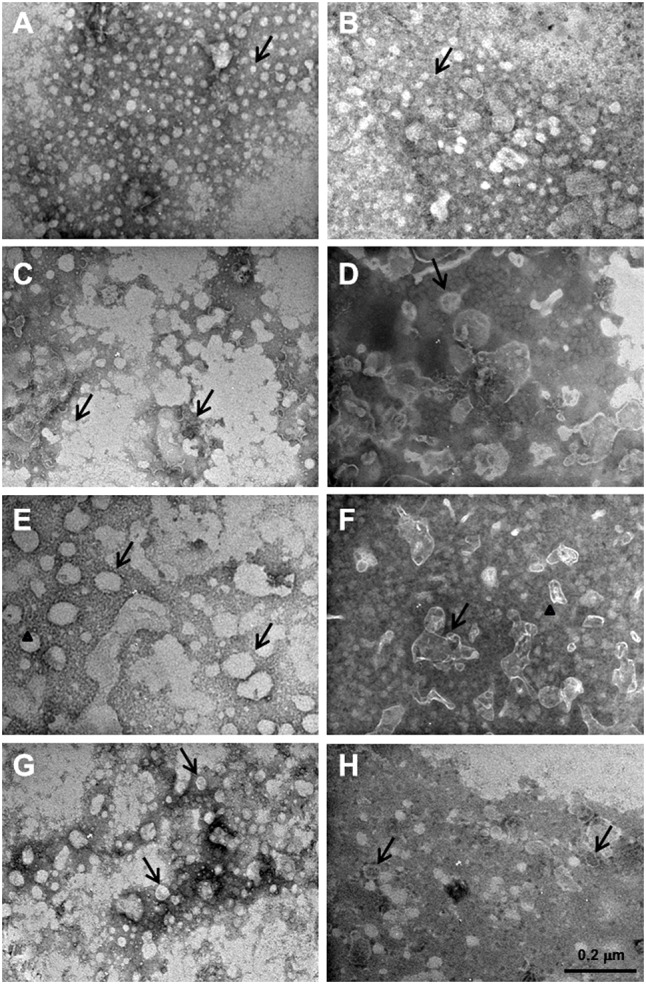
Membrane vesicles of *L. reuteri* DSM 17938 treated with various enzymes. Polymorphic bMVs without an external bilayer (**A**, arrow) and pMVs larger than bMVs (**B**, arrow) after treatment with DNase I; various and broad distributed bMVs (**C**, arrows) and large amount of derived lipid material with irregular shapes and composition obtained by pMVs degradation (**D**, arrows) after treatment with Phospholipase C; merged bMVs (**E**, arrows) and aggregated pMVs (**F**, arrow and arrowhead) in a uniform film treated with Proteinase K; bMVs (**G**, arrows) and pMVs (**H**, arrows) without treatment (controls).

Biofilm membrane vesicles incubated with DNase I yielded small (25–35 nm) polymorphic vesicles without an external bilayer (**Figure 5A**); conversely, pMVs were larger (50–60 nm) than bMVs, but showed a similar polymorphic shape and an external bilayer coating MVs (**Figure 5B**).

Biofilm membrane vesicles incubated with Phospholipase C showed variable and broadly distributed vesicles, with external bilayers (**Figure 5C**, arrows). **Figure 5D** showed the presence of pMVs after treatment with Phospholipase C indicating that treatment degraded the vesicles, and resulted in a large amount of lipids with irregular shapes and composition included in the double membranes. The treatment of bMVs with Proteinase K promoted their fusion (**Figure 5E**, arrows) and resulted in the disappearance of the bilayer structure (**Figure 5E**, arrowhead). Conversely, pMVs were aggregated and formed a uniform film on the external bilayer of MV clusters (**Figure 5F**, arrow and arrowhead). The **Figures 5G,H** showed bMVs and pMVs without any treatment.

## Discussion

The goal of the present study was the detection and physicochemical characterization of MVs from a probiotic strain, as *L. reuteri* DSM 17938, which has been suggested to be effective against infantile colic, alleviation of eczema and *H. pylori* colonization to provide preliminary information about the use of MVs for biotechnological applications. The data demonstrated that *L. reuteri* produces MVs in both planktonic and biofilm phenotypes.

Transmission Electron Microscopy and SEM images showed the MVs were formed by multiple protrusions, similar to those reported for *Bacillus subtilis* (Brown et al., 2014). The size of the *L. reuteri* MVs were in the range from 50–150 nm (**Figure 2**), consistent with the size of MVs reported for other bacteria. MVs of *B. subtilis* for example were similarly distributed, with a mean diameter of 137.7 nm (Brown et al., 2014).

Membrane vesicles generated by Gram-positive bacteria can deliver several virulence factors involved in bacterial pathogenesis. For example, MVs produced by *S. aureus* contain penicillin-binding proteins, which are the target of β-lactam antibiotics, and the membrane-associated global regulator MsrR, which is involved in methicillin resistance (Rossi et al., 2003; Lee et al., 2009; Gurung et al., 2011). MVs help *S. aureus* evade host immune defenses by delivering immunoglobulin G-binding proteins, lipases and super-antigens (Brown et al., 2015). Conversely, MVs of *L. monocytogenes* contain InlB and LLO virulence factors (Lee et al., 2013), which are involved in cellular invasion and escape from vacuoles (Portnoy et al., 1988; Cossart et al., 1989). MVs of *S. pneumoniae* can deliver the toxin pneumolysin (Ply), which is responsible for cell pore formation promoting the colonization and pathogenesis of the bacterium (Hirst et al., 2008; Olaya-Abril et al., 2014). Although the MVs of *C. perfringens* did not have virulence factors, e.g., hemolytic alpha and necrotic enteritis toxin B (NetB) toxins, they contained extracellular and chromosomal DNA. In particular, it was demonstrated the presence of alpha-toxin gene (*plc*), and the perfringolysin O gene (*pfoA*) in MVs isolated by *C. perfringens* (Jiang et al., 2014).

We previously demonstrated that the average size, size distribution (PDI), and Z-potential of OMVs generated from *H. pylori* can be more precisely physicochemical characterized using DLS analysis (Grande et al., 2015). DLS showed that 90% of particles exhibited an average size below 300 nm (hydrodynamic radius). These vesicles are normally distributed and form specific peaks for both phenotypes. The different peak widths of bMVs and pMVs suggested that both planktonic and biofilm MVs are distinct particles, which have a multilayer form, and induce a first- and second-order diffraction consistent with a lamellar bilayer, which are associated with the modification of bilayer asymmetric structure previously demonstrated in other bacterial species (Grande et al., 2015; Jäger et al., 2015). However, the size distribution of bMVs and pMVs were notably distinct. Differences of PDI between bMVs and pMVs may depend on the temperature-dependent liquid to crystalline phase transition of lipids forming the MVs, which can: (i) increase the fluidity of MVs; (ii) promote fusion between natural vesicles; and (iii) modify both average size and size distribution (Jäger et al., 2015).

Differences of average sizes, and size distribution of *L. reuteri* bMVs and pMVs may suggest a potential cargo-sorting effect of molecules making the bilayer structure of MVs similar to that described by Rivera et al. (2010). Notably, bMVs and pMVs contain components that have been shown to mediate communication between bacteria and host, or modulate specific responses *in vitro* and *in vivo* in other bacterial strains (Rivera et al., 2010; Schrempf et al., 2011; Brown et al., 2014; Jiang et al., 2014). For example, different sizes and size distributions depended on the fatty acids and lipid components making up their membranes; and vesicles aggregated or associated with the membranes showed a different electron density depending on the bacterial species and function. The different sizes and size distribution of *L. reuteri* bMVs and pMVs may also stimulate different host responses and affect defense and communications pathways between the host cells and microbiota, thus suggesting a potential messenger activity of bMVs and pMVs.

The Z-potential showed that pMVs and bMVs had a net negative charge consistent with a cell wall charge as previously reported (Grande et al., 2015).

The presence of DNA in MVs has also been previously demonstrated for several Gram-negative bacteria such as *Neisseria gonorrhoeae*, *P. aeruginosa*, *Acinetobacter baumannii*, *H. pylori*, *Haemophilus influenzae*, *Yersinia pestis* and *Shigella flexneri* (Dorward and Garon, 1989; Renelli et al., 2004; Rumbo et al., 2011; Sharpe et al., 2011; Grande et al., 2015). The presence of e_MVs_DNA in Gram-negative bacteria may depend on: (i) the DNA released by lysed cells incorporated by a transformation mechanism (Renelli et al., 2004); (ii) the DNA released in the periplasmic space through OMVs (Kadurugamuwa and Beveridge, 1995); and (iii) the outer inner MVs (O-IMVs) that included DNA, membrane, and cytoplasmatic proteins (Pérez-Cruz et al., 2013).

The association between MVs and eDNA appears to have important consequences in biofilms. eDNA represents a main component of the EPS biofilm matrix in many bacterial species (Grande et al., 2014), and its release depended on several mechanisms, including the excretion of small vesicles from the outer membranes (Renelli et al., 2004; Manning and Kuehn, 2013), Recently, Turnbull et al. (2016) demonstrated that OMVs of *P. aeruginosa*, generated by an “explosive cell lysis mediated through the activity of a cryptic prophage endolysin,” showed nucleic acids and cytoplasmatic proteins in their supramolecular structure. The eDNA EPS-associated in biofilms may support metabolism of sessile cells (Finkel and Kolter, 2001; Mulcahy et al., 2010); maintain the three-dimensional structure of biofilms; promote the horizontal gene transfer (Molin and Tolker-Nielsen, 2003; Spoering and Gilmore, 2006); and bridge the OMV–OMV and OMV–cell interactions (Grande et al., 2015).

PicoGreen staining and the protective effect of MVs on e_MVs_DNA digestion with DNase I indicated that MVs of *L. reuteri* are associated with DNA. This has previously been reported in other Gram-positive bacteria (Jiang et al., 2014; Liao et al., 2014), however, ours is the first to report this in a probiotic species.

Our work suggests that eDNA associated with pMVs and bMVs, with possible implications for structural and biochemical functions of *L. reuteri* DSM 17938.

Interestingly, we found differences between e_MVs_DNA and protein concentrations between bMVs and pMVs, suggesting a different role for eDNA in biofilm and planktonic phenotypes for *L. reuteri* DSM 17938, similar to that as previously reported for *H. pylori* (Grande et al., 2015).

The reaction of MVs with digestive enzymes depended on phenotypes and showed different morphologies (**Figure 5**). The DNase I treatment did not affect the biochemical composition and structure of MVs (**Figures 5A,B**), similar to the results of Chebotar et al. (2013), who demonstrated that MVs of *S. aureus* treated with DNase I did not affect the vesicle structure. Conversely, *L. reuteri* MVs treated with Phospholipase C were degraded demonstrating the importance of phospholipids in MV membrane integrity. Proteinase K treatment data suggest the presence of proteins is involved in the maintenance of MVs structure and have previously been reported for *S. aureus* (Chebotar et al., 2013).

## Conclusion

*Lactobacillus reuteri* DSM 17938 formed MVs both in biofilm and planktonic phenotypes. MVs exhibited the shape, size and composition similar to other biological membranes, and contained e_MVs_DNA, which did not affect the native structure of bMVs and pMVs.

Finally, Fábrega et al. (2016) demonstrated that microbiota vesicles represent a suitable strategy to communicate between beneficial bacteria and intestinal mucosa cells; and MVs could shuttle mediators, that modulated the host immune and defense responses. It will be intriguing to speculate that MVs of *L. reuteri* in the gut microflora are involved in the transport of factors that interact with the host, stimulate the immune system, and activate some factors with antimicrobial activity. If this will be the case, the MVs produced by *L. reuteri* might be considered a safe and biocompatible material to synthesize hybrid immune stimulating nanotherapeutics for customized therapy.

## Author Contributions

RG designed the project, isolated pMVs and bMVs from *L. reuteri*, performed the enzymatic treatment, discussed results and drafted the paper. CC performed the physicochemical characterization of MVs, and the statistical analysis together with LDM. GM, MCDM, LS, and RM performed the PicoGreen experiments, and protein quantifications. AS and MC performed the SEM and TEM analysis. VP performed MVs isolation and MVs biochemical composition together with RG. RS, CC, and ML performed NTA and discussed the results. PS, LH-S, RG, and CC drafted the final editing of paper and critical revised paper.

## Conflict of Interest Statement

The authors declare that the research was conducted in the absence of any commercial or financial relationships that could be construed as a potential conflict of interest.
