# Supplementary material for: Detection and Physicochemical Characterization of Membrane Vesicles (MVs) of Lactobacillus reuteri DSM 17938
Source: Front Microbiol. 2017 Jun 13;8:1040. doi: 10.3389/fmicb.2017.01040 (PMC5468427; doi:10.3389/fmicb.2017.01040)
Supplement: Supplementary file 1 [file Presentation_1.pdf]

Supplementary Information

Detection and Physicochemical Characterization of Membrane Vesicles (MVs) of *Lactobacillus reuteri* DSM 17938.

Rossella Grande[1,2,§*], Christian Celia[1,3,§], Gabriella Mincione[4], Annarita Stringaro[5], Luisa Di Marzio[1], Marisa Colone[5], Maria Carmela Di Marcantonio[4], Luca Savino[4], Valentina Puca[2,6], Roberto Santoliquido[7,8], Marcello Locatelli[1], Raffaella Muraro[4], Luanne Hall-Stoodley[9,10], Paul Stoodley[10,11].

[1]Department of Pharmacy, University of Chieti – Pescara "G. d'Annunzio", Chieti, Italy.

[2] Center for Aging Science and Translational Medicine (CeSI-MeT), Chieti, Italy.

[3]Department of Nanomedicine, Houston Methodist Research Institute, Houston, TX, 77030, USA.

[4]Department of Medical, Oral, and Biotechnological Sciences, University of Chieti – Pescara "G. d'Annunzio", Chieti, Italy.

[5]National Center for Drug Research and Evaluation, Italian National Institute of Health, Rome, Italy.

[6]Department of Biotechnological and Applied Clinical Sciences, University of L'Aquila. L'Aquila, Italy.

[7]AlfatestLab, Cinisello Balsamo (MI), Italy.

[8]Malvern Instruments Ltd., Worcestershire, UK.

[9]NIHR Wellcome Trust Clinical Research Facility, C Level, West Wing, Mailpoint 218, University Hospital Southampton NHS Foundation Trust, Southampton, SO16 6YD, UK;

[10] Department of Microbial Infection and Immunity, Centre for Microbial Interface Biology, The Ohio State University, Columbus, Ohio, 43210 - 2210, USA.

[11]National Center for Advanced Tribology, Faculty of Engineering and the Environment, University of Southampton, Southampton, UK.

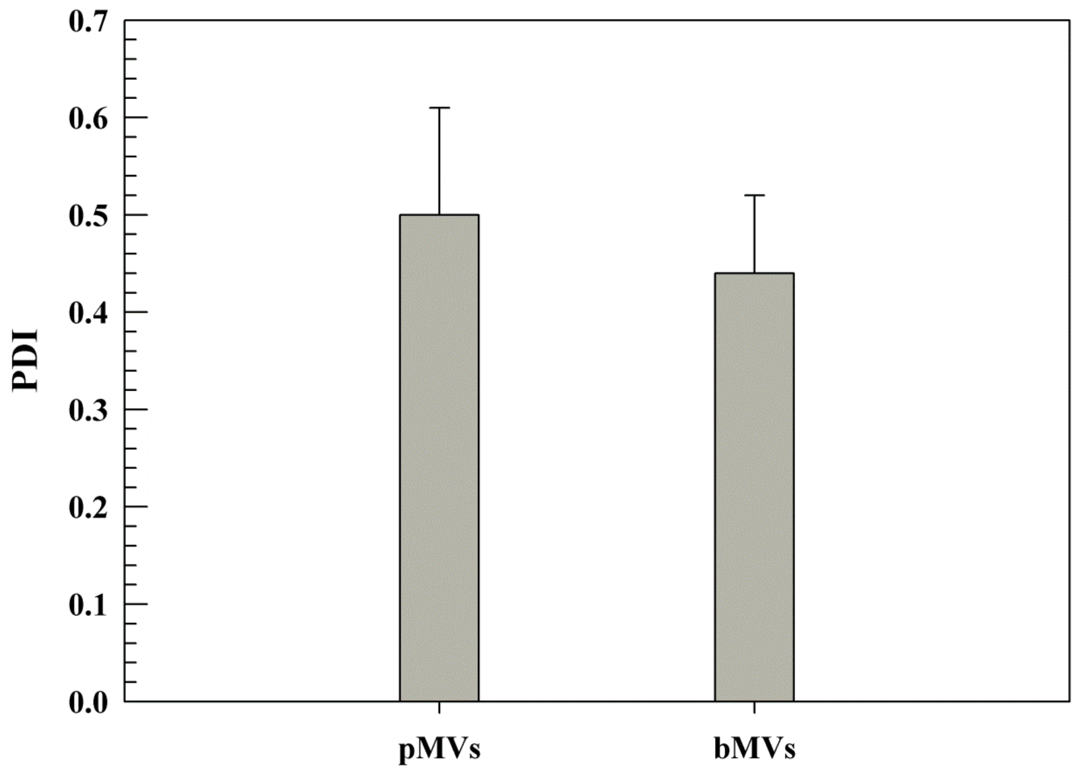

Figure S1. PDI of the pMVs and bMVs of *Lactobacillus reuteri* DSM 17938. The analysis was carried out at 25°C, and data are the average of ten measurements ± standard deviation as triplicates. The error bar if not visible is within the symbol.

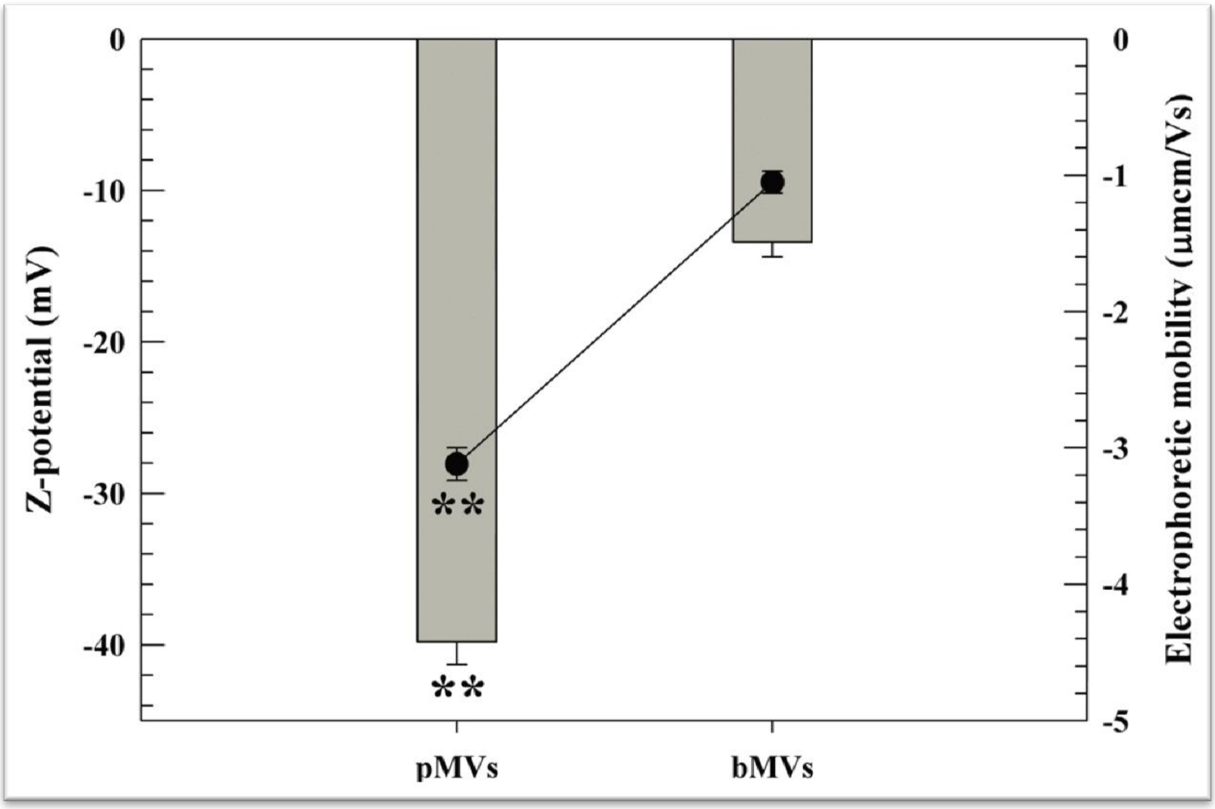

Figure S2. Z – potential and electrophoretic mobility of the pMVs and bMVs of *Lactobacillus reuteri* DSM 17938. The histograms represent the Z – potential values (mV); while the black full circles are the electrophoretic mobility [(µm × cm)/Vs]. The analysis was carried out at 25°C, and data are the average of ten measurements ± standard deviation as triplicates. The error bar if not visible is within the symbol. **$p < 0.01$.

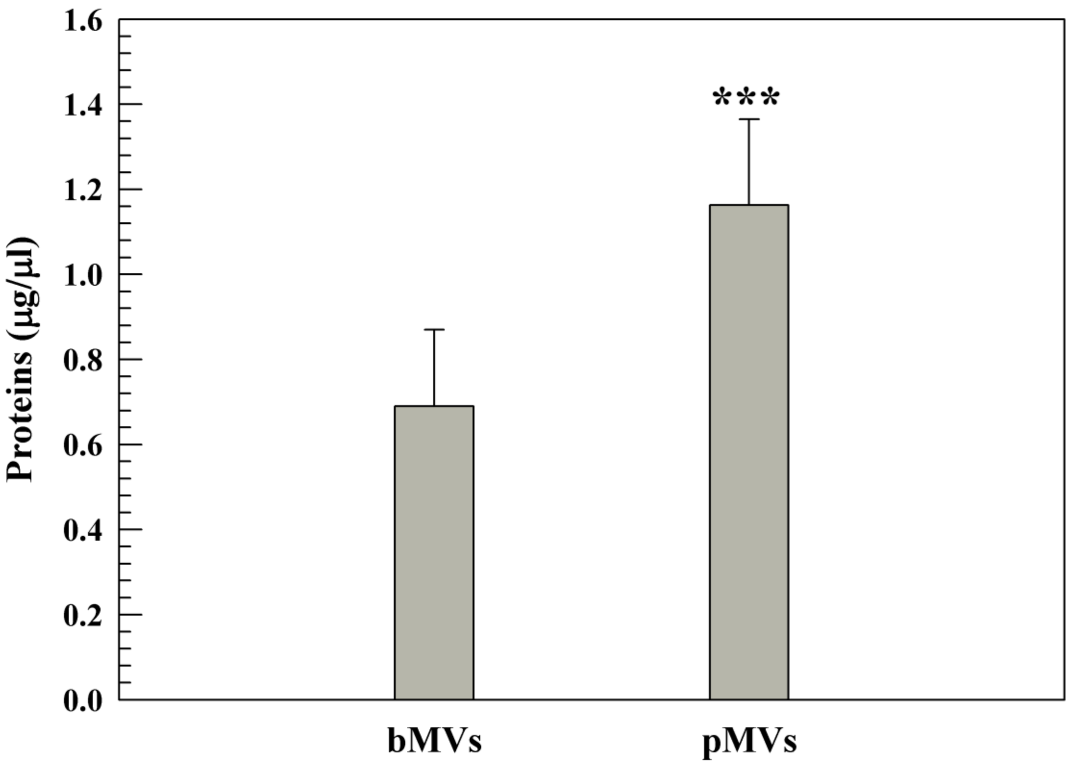

Figure S3. Quantification of proteins associated to pMVs and bMVs. The protein quantification was carried out using the BCA Protein Assay Kit. ***$p \leq 0.001$ compared to pMVs.

Table S1. Averaged tracking analysis of particles versus sizes (nm) for biofilm and planktonic phenotypes of *Lactobacillus reuteri* DSM 17938. D10, D50 and D90 show the average size of 10, 50 and 90% of particles distributed in different samples. The final concentration of bMVs and pMVs was 4.04×10^{10} (particles/ml), and 2.22×10^{10} (particles/ml), respectively. Samples were dispersed in phosphate buffer saline (PBS) before the analysis.

Samples	D10 ± S.E.M.[c]	D50 ± S.E.M.[c]	D90 ± S.E.M.[c]
**bMVs[a]**	62.5 ± 1.2	107.4 ± 2.1	200.6 ± 1.7
**pMVs[b]**	80.1 ± 2.2	129.4 ± 1.1	210.3 ± 1.6

[a]biofilm-associated MVs; [b]planktonic – associated MVs; [c]standard error of the mean (SEM).

Supplementary movie 1: https://figshare.com/articles/Supplementary_Movie_1_avi/4690132

Supplementary movie 2: https://figshare.com/articles/Supplementary_Movie_2/4690141
